# Beneficial HLA-mediated viral polymorphisms on the transmitted virus additively influence disease progression in HIV-1, subtype C infection

**DOI:** 10.1186/1742-4690-9-S2-O60

**Published:** 2012-09-13

**Authors:** RS Ntale, DR Chopera, NK Ngandu, M Abrahams, A Debra, M Mlotswa, L Werner, Z Woodman, K Mlisana, S Abdool Karim, CM Gray, C Williamson

**Affiliations:** 1 Institute of Infectious Disease and Molecular Medicine, UCT, Cape Town, South Africa; 2 National Institute for Communicable Diseases, Johannesburg, South Africa; 3 Centre for AIDS Programme Research in South Africa, UKZN, Durban, South Africa; 4 Department of Molecular and Cell Biology, University of Cape Town, Cape Town, South Africa; 5 Division of Immunology, University of Cape Town , South Africa

## Background

Transmitted viral factors have been shown to affect disease progression but whether infection with viruses carrying beneficial HLA-mediated escape polymorphisms affects disease progression in HLA-mismatched participants remains controversial.

## Methods

Gag was sequenced from 56 participants with acute HIV infection from the CAPRISA 002 cohort. For the newly identified mutation, replication fitness of viruses in PBMCs was compared in a subtype C infectious molecular clone. Disease progression was compared in participants infected with viruses carrying polymorphisms associated with beneficial HLA- B*57/58:01 and B7 supertype (B*39:10/81:01) allelic selective pressure in HLA mismatched participants.

## Results

In HLA-B*57/58:01-negative participants, 51% (25/49) were infected with viruses carrying at least one of the B*57/58:01 fitness cost-associated escape mutations in ISW9 (A146P:19/49), KF11 (A163G: 10/49) or TW10 (T242N/S: 11/49) epitope. In HLA-B7 negative participants, only 9.4% (3/32) had viruses carrying mutations in the HLA-B7 immunodominant TL9 epitope. Furthermore, we identified in viruses from HLA-B7 participants and included in this analysis a novel mutation in Gag p17, Q65H; found in 9.4% (3/32) of B7 negative participants, and with mutant viruses replicating 11% slower than wild-type in tissue culture. Unlike a previous study in this cohort, infection with viruses carrying mutations in HLA-B*57/58:01 restricted epitopes alone did not impact on viral load setpoint in HLA-mismatched participants, although there was a transient trend towards higher CD4 counts at 3 months post infection (p=0.0551). However, HLA-mismatched participants infected with viruses carrying 3 or more HLA footprints associated with fitness cost in Gag had significantly lower viral load and higher CD4 counts at 3 and 12 months post-infection (p=0.028 and p=0.0264, and p=0.0025 and 0.0689).

## Conclusion

These results suggest that multiple mutations generated when viruses are passaged through individuals with beneficial HLAs are needed to attenuate the virus, supporting vaccination methodologies that aim to render the virus less fit.

